# Feasibility of Utilizing Telehealth in a Multidisciplinary Postpartum Hypertension Clinic

**DOI:** 10.1089/whr.2022.0066

**Published:** 2022-11-01

**Authors:** Malamo Countouris, Valentina Jaramillo Restrepo, Shruti Bidani, Janet Catov, Kathryn Berlacher, Arun Jeyabalan, Alisse Hauspurg

**Affiliations:** ^1^ Division of Cardiology, Department of Medicine, University of Pittsburgh Medical Center, Pittsburgh, Pennsylvania, USA.; ^2^ Department of Medicine, University of Pittsburgh Medical Center, Pittsburgh, Pennsylvania, USA.; ^3^ University of Pittsburgh School of Medicine, Pittsburgh, Pennsylvania, USA.; ^4^ Department of Obstetrics, Gynecology, and Reproductive Sciences, University of Pittsburgh, Pittsburgh, Pennsylvania, USA.; ^5^ Department of Epidemiology, University of Pittsburgh, Pittsburgh, Pennsylvania, USA.

**Keywords:** hypertension, hypertensive disorders of pregnancy, preeclampsia, remote monitoring, telemedicine, virtual visits

## Abstract

**Introduction:**

Remote delivery of care improves outcomes following hypertensive disorders of pregnancy, but little is known about the implementation of a multidisciplinary clinic in the virtual space. In this study, we developed a multidisciplinary postpartum hypertension clinic with a telehealth component run jointly with Maternal–Fetal Medicine and Cardiology.

**Materials and Methods:**

Women were referred from Cardiology and Obstetrics providers or through our postpartum remote blood pressure (BP) program and were offered the option of an in-person or telemedicine visit. For virtual visits, BP was recorded by home measurement. We compared clinical and demographic characteristics by visit type (virtual vs. in-person).

**Results:**

Of 175 patients scheduled (2019–2021), 140 attended visits (80% show rate) a mean of 11 weeks postpartum, with 92 (65.7%) seen virtually and 48 (34.2%) seen in-person. Clinical and demographic factors, including self-reported race and insurance type, did not differ between women seen virtually versus in-person. Overall, 97 (69.3%) of women were still on antihypertensive agents at the time of their visit with 33 (34.0%) on more than one antihypertensive agent, which did not differ by visit type. Women who were seen virtually lived a farther distance from the clinic (median 11.6 [interquartile range; IQR 7.7, 30.8] vs. median 7.9 [IQR 5.8, 21.1] miles; *p* = 0.02).

**Conclusions:**

Implementation of a multidisciplinary postpartum hypertension clinic in the virtual space is feasible, targets women at high risk for persistently elevated postpartum BP, and maintains equal attendance compared with in-person visits. Virtual visits deliver care equitably across different racial and socioeconomic groups and may improve access to care in rural areas.

## Introduction

Cardiovascular disease (CVD) is the leading cause of death for women, yet CVD mortality rates among young women remain alarmingly stagnant.^1,2^ Hypertensive disorders of pregnancy (HDP), such as preeclampsia and gestational hypertension, provide a sex-specific window to susceptibility for CVD with a strong association that has been replicated across diverse populations.^3–6^ Women with a history of HDP have excess cardiometabolic risk and progress to overt CVD at younger ages compared with women with uncomplicated pregnancies.^7^

The American Heart Association and the American College of Obstetricians and Gynecologists have identified HDP as a risk factor for CVD with a magnitude of risk on par with smoking and dyslipidemia and have recommended that women with HDP have close follow-up with a cardiologist or primary care physician (PCP) for cardiovascular (CV) risk factor screening and management.^8–10^ The translation of these recommendations into clinical practice has not occurred within most hospital systems, representing a missed opportunity to improve CVD awareness, prevention, and care inequities for women.

In addition to pregnancy providing a critical window to identify women at high risk of developing future CVD, the postpartum period represents a time when women seek health care and are engaged in the system.^11^ As such, interventions in the postpartum period aimed at primary prevention and CV risk factor screening, prevention, and management have the potential to improve health at the individual, community, and system levels. Prior studies have demonstrated the utility of multidisciplinary postpartum hypertension follow-up clinics.^12,13^ A postpartum clinic geared toward caring for women with HDP may have potential benefits of improving follow-up and addressing CV risk factors,^14^ but the optimal structure to maximize feasibility for patients has not been shown. During the COVID-19 pandemic, virtual visits have become an essential option for patients, however, there are limited data on the use of telemedicine in multidisciplinary postpartum hypertension clinics.

**FIG. 1.**
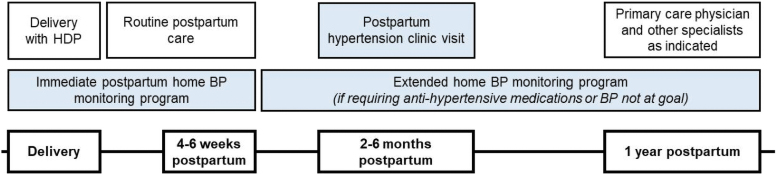
Summary of management of patients with HDP in the postpartum period. Blue shading denotes care that is performed *via* telemedicine. HDP, hypertensive disorders of pregnancy.

In this study, we describe the implementation of a multidisciplinary postpartum hypertension clinic for women with HDP with a telehealth component (Fig. 1). We aimed to (1) establish feasibility of utilizing telehealth in a multidisciplinary postpartum clinic for women with HDP, (2) compare attendance of racial, socioeconomical, and geographically diverse women for both virtual and in-person postpartum appointments, and (3) recruit women at high risk for ongoing postpartum hypertension.

## Materials and Methods

### Clinic development

We developed a multidisciplinary postpartum hypertension clinic run jointly with Maternal–Fetal Medicine (MFM) and Cardiology at the Magee-Womens Hospital (MWH) of the University of Pittsburgh Medical Center (UPMC) in Pittsburgh, PA, USA. As a tertiary care center, MWH cares for a diverse, high-risk population across western and central Pennsylvania with representation from many socioeconomic backgrounds. In addition to a robust obstetrics department, MWH also houses a dedicated Women's Heart Center that is devoted to caring for women with and at risk for heart disease at all stages of life, including pregnancy. To start, we held planning meetings with department leadership, including the Chief of MFM (A.J.) and Director of the Women's Heart Center (K.B). We then additionally held meetings with nursing and operations leadership in MFM and Cardiology to establish the timing, structure, and billing of the clinic.

To inform the standard key providers of postpartum care about the clinic implementation and to encourage referrals, we led departmental Grand Rounds and/or educational sessions with the Departments of Obstetrics, Cardiology, Internal Medicine, Pediatrics, Family Medicine, and Emergency Medicine. These sessions included key stakeholders such as resident physicians, midwives, and nursing staff. Concurrently, we developed patient-facing educational materials, including a brochure (see Supplementary Data), an educational video, and website describing the relationship of HDP to future CVD as well as information about the clinic. Brochures were circulated to patients at the time of their clinical postpartum visit. The educational video was pushed out to patients through our remote blood pressure (BP)-monitoring program.^11^

### Clinic structure

Clinics were held in the afternoon, twice a month, with the capacity to see 10 new patients per clinic in 40-minute visit time slots. We targeted the time period from 6 weeks up to 1 year postpartum for initial visits, but accepted patient referrals for any period postpartum. Because our clinic launched shortly before the start of the pandemic, we underwent a major shift in visit type starting in April 2020, where the majority of our visits were switched to virtual. Virtual visits were conducted through two-way, synchronous interactive telecommunications technology between the patient and the provider. The interactive telecommunication technology included audio and video with the My UPMC application *via* the Vidyo^®^ platform. Patients were instructed to take a home BP before the virtual visit.

Two patients were scheduled for each 40-minute visit and seen sequentially by both a cardiologist and a MFM specialist. For virtual visits, providers took turns entering the virtual room. The MFM specialist focused on debriefing the delivery and HDP diagnosis and discussing contraception, recurrence risk, and preconception considerations as well as routine postpartum concerns, such as breastfeeding and mood disorders. The cardiologist focused on assessing BP control, discussing CV risk, developing a plan for ongoing surveillance and management of CV risk factors, optimizing antihypertensive and other CV medications, and ordering CV imaging or stress testing as appropriate. Patients were recommended to have a screening lipid panel and hemoglobin A1C once they reached at least 3 months after delivery in line with prior studies showing probable normalization of cholesterol and triglyceride levels by that time.^15^

All patient management plans were communicated between MFM and Cardiology providers either verbally or through hospital-system secured network chat application. Each provider documented an individual note for the patient following the visit and a joint consult letter was sent to each patient's obstetrician, PCP, and any other key members of their care team. Each patient was billed as a level 5 single subspecialist visit.

### Data collection

Patient demographics and medical history data were collected *via* manual chart abstraction for each patient seen in the postpartum hypertension clinic (age at clinic visit, height, weight, BP, pregnancy complication, medical history, referrals, laboratory work, prescribed medications) into a RedCap data collection instrument. These patients were matched with the Magee Obstetric Maternal and Infant (MOMI) delivery database for collection of prenatal and pregnancy data (prenatal BPs, prepregnancy body mass index [BMI], gestational age at delivery, birth size, and neonatal ICU admission). The MOMI database was established in 1995 and collects information on maternal, fetal, and neonatal characteristics from admitting services, International Classification of Diseases, Ninth Revision and 10th Revision (ICD-9 and ICD-10) codes, electronic medical record abstraction, electronic birth records, and ultrasound of all women who have delivered at MWH of UPMC.

Chronic hypertension was defined by the American Heart Association/American College of Cardiology 2017 Guidelines.^16^ HDPs were classified according to the American College of Obstetrics and Gynecology 2013 task force on Hypertension in Pregnancy.^17^ Patients were considered to have participated in the remote BP monitoring program if they inputted at least one home BP.^11^ Attendance at postpartum visits with an obstetrician or PCP were abstracted from the electronic medical record. We limited this to individuals seen in the postpartum hypertension clinic up to 6 months before data abstraction performed in March 2021 to allow for assessment of follow-up within 6 months of delivery. We utilized patients' most recent address available in the electronic medical record to compute the travel time and distance to an in-person visit at MWH through a Google Maps Application Programming Interface (API).

Additionally, we obtained area deprivation index (ADI) of the neighborhood residence for each woman at the time of their visit as a proxy for social determinants of health. ADI is a percentile ranking of disadvantage for all locations in the United States based on numerous socioeconomic factors such as income, education, housing, and others.^18,19^ An area in percentile 100 has the greatest level of disadvantage. The ADI dataset was published by the University of Wisconsin and is organized at the fine-grained geography of Census block groups which approximates neighborhood.^20^ We obtained each patient's address from the US Postal Service geocoding API and matched this to ADI using a 9-digit zip code through a crosswalk provided by the Neighborhood Atlas.

Finally, to compare demographic data, clinical characteristics, and follow-up, we collected data on all patients who delivered at MWH with a diagnosis of HDP during the same time period through UPMC Clinical Analytics warehouse, which stores all discrete clinical data documented in the electronic medical record.

### Extension of the postpartum remote monitoring program

We simultaneously developed a remote BP monitoring program for home BP monitoring and management through the first year postpartum as an extension of our 6-week immediate postpartum home BP monitoring program. Our immediate postpartum BP monitoring program has been previously described in detail.^11^ Briefly, patients are enrolled while admitted as inpatients on the postpartum unit or after a postpartum readmission. After discharge from the hospital, women are prompted *via* text message to check their BP daily for the first 2 weeks of the program and between three and five times per week for the remainder of the program through 6 weeks postpartum. BPs are reported into the program *via* text message.

After the first 6 weeks, patients are given the option to continue home monitoring through our extension program (opt-in). The extension program prompts women to report BPs two times per week through the first year postpartum.

### Statistical analyses

We first compared differences in maternal characteristics among women who attended a virtual visit versus those who attended an in-person visit using analysis of variance (ANOVA) for continuous variables and chi-squared tests for categorical variables. Then, we compared differences in maternal demographic characteristics of patients seen in our postpartum hypertension clinic versus all those with a diagnosis of HDP in our medical system over the same time period. We used Fisher's exact tests for expected counts that were less than 5. We compared non-normally distributed continuous variables using a rank sum test. *p*-Values <0.05 were considered statistically significant. Statistical analyses were conducted using Stata version 13.0. This study was approved by the University of Pittsburgh Institutional Review Board (STUDY21010100).

## Results

Out of 175 scheduled visits (December 2019 through April 2021), 140 patients were seen in the clinic, corresponding with a show rate of 80.0%. On further breakdown by visit type, 122 of visits were scheduled virtually with a 75.4% show rate and 53 visits were scheduled in-person with a 90.6% show rate. Of 19 clinics during this time period with full capacity to see 10 patients, we filled 71% of the possible clinic slots.

Complete demographic data from patients seen in the postpartum hypertension clinic are shown in Tables 1 and 2 and compared by visit type (virtual vs. in-person). Of the women seen in our clinic, 32.9% were of Black race, 37.8% were on medical assistance, and mean BMI was in the obese range (31.9 ± 8.7 kg/m^2^). For HDP type, 20.7% had gestational hypertension, 48.6% had preeclampsia, and 25.7% had superimposed preeclampsia on chronic hypertension. Overall, 39.3% of women delivered preterm (<37 weeks gestation).

**Table 1.** Prepregnancy Demographic Characteristics for Patients Seen Virtually Compared with In-Person in the Postpartum Hypertension Clinic

	Overall cohort (*N* = 140)	Virtual visit (*N* = 92)	In-person visit (*N* = 48)	*p*
Prepregnancy demographics				
Age (years)^a^	33.6 ± 5.7	33.1 ± 5.7	34.6 ± 5.5	0.14
Race, *n* (%)				0.36
Asian	9 (6.4)	4 (4.4)	5 (10.2)	
Black	46 (32.9)	28 (30.4)	18 (37.5)	
White	82 (58.6)	57 (62.0)	25 (52.1)	
None of the above	3 (2.1)	3 (3.3))	0 (0)	
Hispanic ethnicity, *n* (%)	3 (2.1)	0 (0)	3 (6.3)	0.04
Type of insurance, *n* (%)				0.77
Private	84 (60.0)	56 (60.9)	28 (58.3)	
Medicaid	56 (40.0)	36 (39.1)	20 (41.7)	
ADI	64.5 (25.0)	62.0 (24.7)	69.2 (24.9)	0.1
Distance from clinic, median [IQR] (miles)	11.3 [6.1, 25]	11.6 [7.7, 30.8]	7.9 [5.8, 21.1]	0.02
Prepregnancy BMI (kg/m^2^)	31.9 ± 8.7	32.3 ± 8.1	31.1 ± 9.6	0.46
First prenatal systolic BP (mmHg)	123.1 ± 13.2	122.6 ± 13.3	124.2 ± 13.1	0.51
First prenatal diastolic BP (mmHg)	75.1 ± 9.2	75.8 ± 8.3	73.7 ± 10.6	0.24

^a^ Data are mean ± SD unless otherwise specified.

ADI, area deprivation index; BMI, body mass index; BP, blood pressure; IQR, interquartile range; SD, standard deviation.

**Table 2.** Pregnancy Outcomes and Postpartum Follow Up for Patients Seen Virtually Compared with In-Person in the Postpartum Hypertension Clinic

	Overall cohort (*N* = 140)	Virtual visit (*N* = 92)	In-person visit (*N* = 48)	*p*
Pregnancy outcomes				
Hypertension in pregnancy diagnosis, *n* (%)				0.07
Chronic hypertension	7 (5.0)	3 (3.3)	4 (8.3)	
Gestational hypertension	29 (20.7)	24 (27.0)	5 (11.4)	
Preeclampsia	68 (48.6)	45 (50.6)	23 (52.3)	
Superimposed preeclampsia	36 (25.7)	20 (22.5)	16 (36.4)	
Peripartum cardiomyopathy, *n* (%)	8 (5.7)	6 (6.5)	2 (4.2)	0.57
Gestational diabetes, *n* (%)	18 (13.5)	12 (13.8)	6 (13.0)	0.90
Preterm delivery, *n* (%)	55 (39.3)	37 (40.2)	18 (37.5)	0.76
Fetal characteristics				
Gestational age at delivery (weeks)^a^	36.5 ± 3.6	36.8 ± 3.2	35.9 ± 4.4	0.20
Small for gestational age, *n* (%)	23 (18.4)	11 (13.4)	12 (27.9)	0.12
Neonatal ICU admission, *n* (%)	36 (28.8)	23 (28.1)	13 (30.2)	0.80
Follow-up				
OB postpartum visit, *n* (%)	128 (91.4)	86 (93.5)	42 (87.5)	0.23
PCP visit, *n* (%)	38 (39.2)	23 (37.7)	15 (41.7)	0.70
Remote BP program (≤ 6 weeks PP), *n* (%)	124 (88.6)	84 (91.3)	40 (83.3)	0.16
Extended remote BP program (> 6 weeks PP)^b^, *n* (%)	14 (35.9)	10 (35.7)	4 (36.4)	0.97

^a^ Data are mean ± SD unless otherwise specified.

^b^ Restricted to delivery date after September 1, 2020.

OB, obstetrician; PCP, primary care physician; PP, postpartum.

**FIG. 2.**
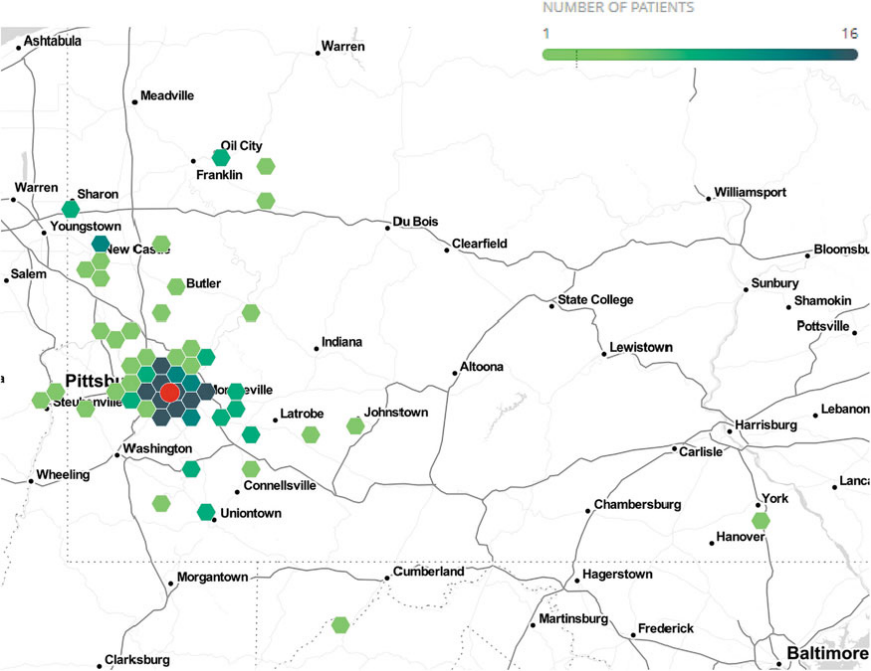
Geographic distribution of patients seen based on home address. Location of Magee-Womens Hospital indicated by red circle.

Women seen at a virtual visit were less likely to be of Hispanic ethnicity (0.0% vs. 6.3%; *p* = 0.04) and lived further from the clinic (median 11.6 [IQR 7.7, 30.8] vs. 7.9 [IQR 5.8, 21.1] miles; *p* = 0.02) compared with those seen at an in-person visit. The broad geographic distribution of all patients seen in the clinic is shown in Figure 2. Otherwise, there were no differences in demographic characteristics, including age, self-reported race, type of insurance, ADI, nor pregnancy complications or postpartum follow-up by visit type.

Women were seen in the postpartum hypertension clinic at mean of 11 weeks (76.7 ± 63.0 days) after their delivery (Table 3). The primary referral source for the clinic was from obstetricians (55.0%). Most women participated in immediate postpartum remote home BP monitoring (88.6%) with 35.9% continuing monitoring beyond 6 weeks postpartum. A majority (69.3%) of women were on antihypertensive medications at the time of their clinic visit, with 34.0% on more than one antihypertensive agent. BPs on the day of clinic visit were well controlled with a mean of 126 ± 15/82 ± 11 mmHg.

**Table 3.** Postpartum Hypertension Clinic Cohort Characteristics, Management, and Referrals by Visit Type

	Overall cohort (*N* = 140)	Virtual visit (*N* = 92)	In-person visit (*N* = 48)	*p*
Clinic cohort characteristics				
Time from delivery to clinic visit (days)^a^	76.7 ± 63.0	74.6 ± 55.7	80.6 ± 75.3	0.60
Referral source to clinic, *n* (%)				0.19
Obstetrician or inpatient obstetrics team	77 (55.0)	49 (53.2)	28 (58.3)	
Inpatient cardiology team	25 (17.9)	13 (14.3)	12 (25.0)	
Self-referred or remote monitoring program	8 (5.7)	6 (6.5)	2 (4.2)	
Other or unknown	30 (21.4)	24 (26.1)	6 (12.5)	
Systolic BP (mmHg)	126 ± 15	125 ± 15	128 ± 15	0.22
Diastolic BP (mmHg)	82 ± 11	81 ± 11	83 ± 10	0.18
On antihypertensive agents, *n* (%)	97 (69.3)	61 (66.3)	36 (75.0)	0.29
On >1 antihypertensive agent, *n* (%)	33 (34.0)	21 (34.4)	12 (33.3)	0.91
BMI at time of visit (kg/m^2^)	31.2 ± 8.1	31.6 ± 7.3	30.3 ± 9.6	0.42
Addressed contraception, *n* (%)	133 (95.0)	87 (94.6)	46 (95.8)	0.74
Traditional cardiovascular risk factors				
Obese (BMI ≥30 kg/m^2^), *n* (%)	61 (48.0)	43 (50.0)	18 (43.9)	0.52
Tobacco use, *n* (%)				0.93
Current	16 (11.6)	11 (12.1)	5 (10.6)	
Former	28 (20.3)	19 (20.9)	9 (19.2)	
Prepregnancy hypertension, *n* (%)	46 (32.9)	25 (27.2)	21 (43.8)	0.047
Diabetes, *n* (%)	6 (4.3)	4 (4.4)	2 (4.2)	1.0
Family history of early CVD, *n* (%)	23 (16.6)	14 (15.4)	9 (18.8)	0.61
Cardiovascular risk factor screening				
Postpartum lipid panel completed, *n* (%)	74 (52.9)	47 (51.1)	27 (56.3)	0.56
Time from clinic visit to lipid panel measurement [IQR] (days)	148 [40, 378]	154 [51, 426]	132 [0, 250]	0.24
Total cholesterol, median [IQR] (mg/dL)	183 [155, 201]	184 [150, 201]	181 [157, 205]	0.70
HDL, median [IQR] (mg/dL)	55 [46, 69]	54 [46, 64]	55 [42, 73]	0.87
LDL, median [IQR] (mg/dL)	99 [82, 123]	97 [76, 118]	100 [84, 134]	0.35
Postpartum HbA1C completed, *n* (%)	60 (42.9)	39 (42.4)	21 (43.8)	0.88
HbA1C, median [IQR] (%)	5.3 [5.1, 5.7]	5.4 [4.9, 5.8]	5.2 [5.1, 5.4]	0.46
Echocardiogram, *n* (%)	50 (35.7)	30 (32.6)	20 (41.7)	0.29
Left ventricular EF (%)	55 ± 11	55 ± 12	56 ± 8	0.60
EF <50%, *n* (%)	8 (16)	6 (20.0)	2 (10.0)	0.35
Estimated PA systolic pressure (mmHg)	34 ± 10	32 ± 8	37 ± 13	0.28
Referrals from postpartum hypertension clinic				
Nutritionist, *n* (%)	37 (26.4)	25 (27.2)	12 (25.0)	0.78
Sleep medicine, *n* (%)	9 (6.4)	7 (7.6)	2 (4.2)	0.72
Psychiatry, *n* (%)	35 (25.0)	23 (25.0)	12 (25.0)	1.0

^a^ Data are mean ± SD unless otherwise specified.

CVD, cardiovascular disease; EF, ejection fraction; HbA1C, hemoglobin A1C; HDL, high-density lipoprotein; LDL, low-density lipoprotein; PA, pulmonary artery.

Women seen for an in-person visit were more likely to have a diagnosis of prepregnancy hypertension (43.8% vs. 27.2%, *p* = 0.047), but otherwise had no differences in BP, CV risk factors, CV risk factor screening, or referrals by visit type (Table 3). Slightly over half (53%) of women seen had a screening lipid panel, obtained a median of 148 days (21 weeks) after their clinic visit date.

When compared with the overall population of patients with a HDP who delivered at MWH during the same time frame (*n* = 2307), patients seen in the postpartum hypertension clinic were older, more likely to identify as Black race and were equally likely to be insured through medical assistance (Table 4). Additionally, women seen in the postpartum hypertension clinic were a high-risk group, more likely to have an Emergency Room visit (18.6% vs. 11.9%, *p* = 0.02) or hospital readmission within 8 weeks postpartum (13.6% vs. 4.4%, *p* < 0.01) and were more likely to attend a follow-up visit with their PCP in the first year postpartum (43.3% vs. 29.2%, *p* < 0.01).

**Table 4.** Select Demographic and Follow-Up Characteristics for Patients Seen in the Postpartum Hypertension Clinic Compared with Overall Deliveries Complicated by Hypertensive Disorders of Pregnancy

	Overall deliveries with HDP^a^ (*N* = 2307)	Seen in HDP clinic (*N* = 140)	*p*
Pregnancy demographics			
Age, mean ± SD (years)^b^	30.0 ± 5.9	33.6 ± 5.7	<0.01
Race, *n* (%)			0.02
White	1616 (70.0)	82 (58.6)	
Black	551 (23.9)	46 (32.9)	
Asian	81 (3.5)	9 (6.4)	
Other	59 (2.6)	3 (2.1)	
Type of insurance, *n* (%)			0.8
Private	1404 (60.9)	84 (60.0)	
Medicaid	903 (39.1)	56 (40.0)	
ADI	63.1 (26.4)	64.5 (25.0)	0.5
Follow-up characteristics			
OB postpartum visit, *n* (%)	2020 (87.6)	128 (91.4)	0.2
PCP visit, *n* (%)	674 (29.2)	60 (43.3)	<0.01
Emergency room visit postpartum, *n* (%)	275 (11.9)	26 (18.6)	0.02
Hospital readmission postpartum, *n* (%)	101 (4.4)	19 (13.6)	<0.01

^a^ Deliveries at MWH during same time period complicated by a hypertensive disorder of pregnancy.

^b^ Data are mean ± SD unless otherwise specified.

HDP, hypertensive disorders of pregnancy; MWH, Magee-Womens Hospital.

## Discussion

In this study, we demonstrate feasibility of utilizing telehealth in a postpartum hypertension clinic targeting women with HDP. When comparing women seen virtually versus in-person, we found no differences by race or insurance type. Women seen virtually lived further from the clinic and had no differences in ADI from those seen in-person. Most women seen in our clinic were at high risk for postpartum hypertension with over 69% on antihypertensive medications at the time of their visit. Additionally, when compared with our overall population of women with HDP, women seen in the clinic were more racially diverse, were equally likely to be insured through Medicaid, and likely had higher postpartum morbidity as suggested by the higher rates of emergency room visits and hospital readmissions after delivery.

There are a number of potential benefits of leveraging virtual visits for follow-up care for women in the postpartum period or “fourth trimester.” Prior studies have identified time, being too busy with childcare, work, and difficulty attending appointments as major barriers to follow-up.^21,22^ The attendance rate in our clinic (80%) exceeds the national average of postpartum visit attendance, which has been reported to be ∼60% in prior studies.^23^ A virtual model may be more feasible for new mothers while caring for a newborn at home and have an advantage in that women can attend in the comfort of their own home and often without the need for alternative childcare. This is especially relevant in the midst of a pandemic like COVID-19, where women may be concerned about infection risks coming into the clinic.^24^

Our clinic model is one that can be adopted at health care centers across the country, particularly to improve postpartum hypertension care in nonurban areas, as telehealth plays an important role in improving access to care in these locations. Postpartum adherence to recommended follow-up has been previously shown to be lower in nonurban areas, likely due to geographic isolation, lack of access to reliable transportation, and lower socioeconomic status. Other identified barriers include lack of childcare and rapid return to work after delivery.^25^ A lack of access to high-quality maternal health services in rural communities is the result of many factors, including hospital and obstetric department closures, workforce shortages, and access to care challenges arising from the social determinants of health, which have contributed to disparities in maternal health care for rural women.^25^ Fewer than 50% of rural women have access to perinatal services within a 30-minute drive of their home and more than 10% of rural women drive over 100 miles for these services.^26^

Although prior studies have cited concerns about access to telemedicine visits in rural locations,^27^ we showed that women who attended virtual visits lived further from the clinic, demonstrating a preference for virtual visits among women who live further away. Telemedicine and telehealth services have also been successfully implemented in rural settings across multiple fields of medicine.^28^ An active area of clinical work for our group is expansion of our clinic to more rural locations within the UPMC system, including our first satellite location in Erie, Pennsylvania.

We demonstrate that women of diverse backgrounds and socioeconomic status are equally likely to attend virtual and in-person visits. More specifically, we saw no difference in virtual visit attendance by self-reported race, insurance type, or ADI, raising the possibility that virtual visits are an opportunity to improve access and equity for high-risk women following pregnancy. Although we saw few Hispanic patients (2% of our population), this is consistent with population demographics for the Pittsburgh area (3% Hispanic).^29^ Hispanic patients were more likely to be seen for in-person visits possibly due to easier incorporation of translator services for the in-person visits. In the future, improving incorporation of remote translator services may be beneficial for facilitation of virtual visits for non-English-speaking patients.

The role of telehealth has been critical in the development and success of our clinic's model and the utilization of a remote BP monitoring program allowed for timely assessments and management of hypertension. Women are often unaware of the association between HDP and future CV risk and postpartum BP control is suboptimal. Prior studies have found that over 40% of women with a history of severe preeclampsia still had significant hypertension 1 year after delivery and those who were on therapy did not have optimal control.^30^ Of women who are overweight and obese, 60% develop chronic hypertension by 1 year postpartum.^31^

Importantly, our clinic successfully targeted women at higher risk for development of hypertension after pregnancy. While a large majority of women seen in the clinic remained on antihypertensive medications, they had adequately controlled BPs. In addition, mean BPs were similar among women seen virtually versus in-person. This may be attributed to the success of our postpartum remote BP monitoring program, which allows for management and timely titration of medications from home for optimization of BP control within the weeks and months postpartum.

Women with HDP also have increased cardiometabolic risk in just the year postpartum with higher likelihood of dyslipidemia and higher cardiovascular risk scores,^32^ as supported by the high percentage of comorbid CV risk factors among women seen in our clinic. Nearly 50% of women seen in our clinic had a BMI in the obese range (≥30 kg/m^2^) warranting discussion of lifestyle modifications for weight loss. About a quarter of patients seen in our clinic were referred to see a nutritionist, free of charge, and about 6% were referred for sleep apnea evaluation.

This highlights the potential benefits of a multidisciplinary postpartum hypertension clinic and relevance of focusing on CV risk factor management and CVD prevention for this at-risk population as supported by prior studies.^33^ This vital counseling, education, and management can occur both in-person or virtually based on our clinic data. They also highlight the critical need for universal expansion of Medicaid coverage to at least 1 year postpartum. Future goals of our clinic include more standardized approaches to referrals and assessing adherence with scheduled specialist visits.

Despite the almost unanimous recommendation and ordering of lipid panel testing at the clinic visit, only 53% of patients seen in our clinic ultimately had a screening lipid panel drawn. To address this, we plan to implement home test kits to improve the number of women obtaining recommended lipid panel screening. Further investigation is needed to identify how to address barriers in obtaining screening, such as a lipid panel, for traditional cardiovascular risk score estimation in the postpartum period and to ensure that those who are seen remotely have equal screening tests ordered.^34^

Our study demonstrates that postpartum hypertension clinics can help bridge care from the obstetricians to internists who would follow patients longitudinally. Importantly, by having patients see an MFM specialist in our clinic, patients have the opportunity to address any unmet obstetrics needs and questions about future pregnancies. We demonstrate higher rates of follow-up with PCPs in our cohort (43%) compared with prior reports and when compared with our source population (25%–29%).^35,36^ This is possibly related to the direct referral system we have incorporated into the clinic, but we cannot exclude the possibility of selection bias. Future studies are needed to assess the effect of a postpartum hypertension clinic on longer term outcomes, including follow-up beyond 1 year postpartum, BP management, longitudinal cardiovascular risk factor screening, and modification of cardiovascular risk factors.

This study has a few limitations. First, it is a single-center study, which may limit its generalizability to other centers, however, as noted above, we saw a geographically and racially diverse population. This clinic model is built on the presence of two subspecialists, which may not be financially or logistically feasible at nontertiary care centers. Alternative models could consider a visit with a cardiologist or primary care provider who is well versed in obstetric and contraceptive care. There was also a limited follow-up window, since clinic visits commenced in December of 2019. Because data were abstracted from our health system's electronic medical record, we may have missed follow-up visits for those outside our health system. Future studies with patients followed longitudinally would better assess the proportion of patients seen by PCPs in follow-up. We were unable to account for breastfeeding and its impact on BP and lipid levels due to lack of survey data on breastfeeding. Patient scheduling was limited by provider and clinic availability.

We could not account for patients who rescheduled visits and thus left unfilled appointment slots at the last minute. Finally, our center is uniquely equipped with a robust remote BP monitoring and management program, which likely contributed to high patient engagement and institutional support of the clinic, which may not be the case at all sites. Future studies would be helpful to investigate the number of women who received medication titration or adjustment through the program.

## Conclusions

In conclusion, we have found that implementation of a multidisciplinary postpartum hypertension follow-up clinic utilizing telemedicine is feasible, targets women at high risk for persistently elevated postpartum BPs, and maintains equal attendance compared with in-person visits. Virtual visits deliver care equitably across a racially and socioeconomically diverse population and may improve access to care in rural areas.

## Supplementary Material

Supplemental data

## Abbreviations Used

ADI: area deprivation index
API: Application Programming Interface
BMI: body mass index
BP: blood pressure
CV: cardiovascular
CVD: cardiovascular disease
HbA1C: hemoglobin A1C
HDP: hypertensive disorders of pregnancy
HDL: high-density lipoprotein
IQR: interquartile range
LDL: low-density lipoprotein
MFM: Maternal–Fetal Medicine
MOMI: Magee Obstetric Maternal and Infant
MWH: Magee-Womens Hospital
PCP: primary care physician
SD: standard deviation
UPMC: University of Pittsburgh Medical Center

## References

[1] Arora S, Stouffer GA, Kucharska-Newton AM, et al. Twenty year trends and sex differences in young adults hospitalized with acute myocardial infarction. Circulation 2019;139(8):1047–1056. 30586725 10.1161/CIRCULATIONAHA.118.037137 PMC6380926

[2] Mozaffarian D, Benjamin EJ, Go AS, et al. Heart disease and stroke statistics—2015 update: A report from the American Heart Association. Circulation 2015;131(4):e29–e322. 25520374 10.1161/CIR.0000000000000152

[3] Wu P, Haththotuwa R, Kwok CS, et al. Preeclampsia and future cardiovascular health: A systematic review and meta-analysis. Circ Cardiovasc Qual Outcomes 2017;10(2):e003497. 28228456 10.1161/CIRCOUTCOMES.116.003497

[4] Bellamy L, Casas JP, Hingorani AD, et al. Pre-eclampsia and risk of cardiovascular disease and cancer in later life: Systematic review and meta-analysis. BMJ 2007;335(7627):974. 17975258 10.1136/bmj.39335.385301.BE PMC2072042

[5] Hauspurg A, Ying W, Hubel CA, et al. Adverse pregnancy outcomes and future maternal cardiovascular disease. Clin Cardiol 2018;41(2):239–246. 29446836 10.1002/clc.22887 PMC6490154

[6] McDonald SD, Malinowski A, Zhou Q, et al. Cardiovascular sequelae of preeclampsia/eclampsia: A systematic review and meta-analyses. Am Heart J 2008;156(5):918–930. 19061708 10.1016/j.ahj.2008.06.042

[7] Langlois AWR, Park AL, Lentz EJM, et al. Preeclampsia brings the risk of premature cardiovascular disease in women closer to that of men. Can J Cardiol 2020;36(1):60–68. 31735430 10.1016/j.cjca.2019.06.028

[8] Mosca L, Benjamin EJ, Berra K, et al. Effectiveness-based guidelines for the prevention of cardiovascular disease in women—2011 update: A guideline from the American Heart Association. J Am Coll Cardiol 2011;57(12):1404–1423. 21388771 10.1016/j.jacc.2011.02.005 PMC3124072

[9] ACOG Practice Bulletin No. 202: Gestational hypertension and preeclampsia. Obstet Gynecol 2019;133(1):1. 10.1097/AOG.0000000000003018 30575675

[10] Parikh NI, Gonzalez JM, Anderson CAM, et al. Adverse pregnancy outcomes and cardiovascular disease risk: Unique opportunities for cardiovascular disease prevention in women: A scientific statement from the American Heart Association. Circulation 2021;143(18):e902–e916. 33779213 10.1161/CIR.0000000000000961

[11] Hauspurg A, Lemon LS, Quinn BA, et al. A postpartum remote hypertension monitoring protocol implemented at the hospital level. Obstet Gynecol 2019;134(4):685–691. 31503166 10.1097/AOG.0000000000003479 PMC7289450

[12] Cusimano MC, Pudwell J, Roddy M, et al. The maternal health clinic: An initiative for cardiovascular risk identification in women with pregnancy-related complications. Am J Obstet Gynecol 2014;210(5):438.e431–e439. 10.1016/j.ajog.2013.12.001 24316270

[13] Celi AC, Seely EW, Wang P, et al. Caring for women after hypertensive pregnancies and beyond: Implementation and integration of a postpartum transition clinic. Matern Child Health J 2019;23(11):1459–1466. 31257555 10.1007/s10995-019-02768-7

[14] Gladstone RA, Pudwell J, Pal RS, et al. Referral to cardiology following postpartum cardiovascular risk screening at the Maternal Health Clinic in Kingston, Ontario. Can J Cardiol 2019;35(6):761–769. 31151712 10.1016/j.cjca.2019.03.008

[15] Wiznitzer A, Mayer A, Novack V, et al. Association of lipid levels during gestation with preeclampsia and gestational diabetes mellitus: A population-based study. Am J Obstet Gynecol 2009;201(5):482.e481–e488. 10.1016/j.ajog.2009.05.032 PMC5483324 19631920

[16] Whelton PK, Carey RM, Aronow WS, et al. 2017 ACC/AHA/AAPA/ABC/ACPM/AGS/APhA/ASH/ASPC/NMA/PCNA guideline for the prevention, detection, evaluation, and management of high blood pressure in adults: A report of the American College of Cardiology/American Heart Association Task Force on Clinical Practice Guidelines. Hypertension 2018;71(6):e13–e115. 29133356 10.1161/HYP.0000000000000065

[17] American College of Obstetricians, Gynecologists, Task Force on Hypertension in Pregnancy. Report of the American College of Obstetricians and Gynecologists' Task Force on Hypertension in Pregnancy. Obstet Gynecol 2013;122(5):1122–1131. 24150027 10.1097/01.AOG.0000437382.03963.88

[18] Kind AJH, Buckingham WR. Making neighborhood-disadvantage metrics accessible—The neighborhood atlas. N Engl J Med 2018;378(26):2456–2458. 29949490 10.1056/NEJMp1802313 PMC6051533

[19] Singh GK. Area deprivation and widening inequalities in US mortality, 1969–1998. Am J Public Health 2003;93(7):1137–1143. 12835199 10.2105/ajph.93.7.1137 PMC1447923

[20] Neighborhood Atlas. Available from: https://www.neighborhoodatlas.medicine.wisc.edu/ [Last accessed: December 16, 2020].

[21] Chan SE, Nowik CM, Pudwell J, et al. Standardized postpartum follow-up for women with pregnancy complications: Barriers to access and perceptions of maternal cardiovascular risk. J Obstet Gynaecol Can 2021;43(6):746–755. 33766754 10.1016/j.jogc.2021.03.006

[22] Bennett WL, Ennen CS, Carrese JA, et al. Barriers to and facilitators of postpartum follow-up care in women with recent gestational diabetes mellitus: A qualitative study. J Womens Health (Larchmt) 2011;20(2):239–245. 21265645 10.1089/jwh.2010.2233 PMC3064871

[23] McKinney J, Keyser L, Clinton S, et al. ACOG Committee Opinion No. 736: Optimizing postpartum care. Obstet Gynecol 2018;132(3):784–785. 30134408 10.1097/AOG.0000000000002849

[24] Fryer K, Delgado A, Foti T, et al. Implementation of obstetric telehealth during COVID-19 and beyond. Matern Child Health J 2020;24(9):1104–1110. 32564248 10.1007/s10995-020-02967-7 PMC7305486

[25] Centers for Medicare and Medicaid Services Improving access to maternal health care in rural communities; 2019. Available from: https://www.cms.gov/About-CMS/Agency-Information/OMH/equity-initiatives/rural-health/09032019-Maternal-Health-Care-in-Rural-Communities.pdf [Last accessed: August 18, 2021].

[26] Hung P, Henning-Smith CE, Casey MM, et al. Access to obstetric services in rural counties still declining, with 9 percent losing services, 2004-14. Health Aff (Millwood) 2017;36(9):1663–1671. 28874496 10.1377/hlthaff.2017.0338

[27] Woodall T, Ramage M, LaBruyere JT, et al. Telemedicine services during COVID-19: Considerations for medically underserved populations. J Rural Health 2021;37(1):231–234. 32613657 10.1111/jrh.12466 PMC7364549

[28] Kane-Gill SL, Rincon F. Expansion of telemedicine services: Telepharmacy, telestroke, teledialysis, tele-emergency medicine. Crit Care Clin 2019;35(3):519–533. 31076051 10.1016/j.ccc.2019.02.007

[29] United States Census Bureau. Available from: https://www.census.gov/quickfacts/pittsburghcitypennsylvania [Last accessed: August 2, 2021].

[30] Benschop L, Duvekot JJ, Versmissen J, et al. Blood pressure profile 1 year after severe preeclampsia. Hypertension 2018;71(3):491–498. 29437895 10.1161/HYPERTENSIONAHA.117.10338

[31] Hauspurg A, Countouris ME, Jeyabalan A, et al. Risk of hypertension and abnormal biomarkers in the first year postpartum associated with hypertensive disorders of pregnancy among overweight and obese women. Pregnancy Hypertens 2019;15:1–6. 30825904 10.1016/j.preghy.2018.10.009 PMC6400304

[32] Moe K, Sugulle M, Dechend R, et al. Risk prediction of maternal cardiovascular disease one year after hypertensive pregnancy complications or gestational diabetes mellitus. Eur J Prev Cardiol 2020;27(12):1273–1283. 31600083 10.1177/2047487319879791

[33] Hauspurg A, Countouris ME, Catov JM. Hypertensive disorders of pregnancy and future maternal health: How can the evidence guide postpartum management? Curr Hypertens Rep 2019;21(12):96. 31776692 10.1007/s11906-019-0999-7 PMC7288250

[34] Yuan N, Pevnick JM, Botting PG, et al. Patient use and clinical practice patterns of remote cardiology clinic visits in the era of COVID-19. JAMA Netw Open 2021;4(4):e214157. 33818619 10.1001/jamanetworkopen.2021.4157 PMC8022216

[35] Spaan J, Peeters L, Spaanderman M, et al. Cardiovascular risk management after a hypertensive disorder of pregnancy. Hypertension 2012;60(6):1368–1373. 23071130 10.1161/HYPERTENSIONAHA.112.198812

[36] Lewey J, Levine LD, Yang L, et al. Patterns of postpartum ambulatory care follow-up care among women with hypertensive disorders of pregnancy. J Am Heart Assoc 2020;9(17):e016357. 32851901 10.1161/JAHA.120.016357 PMC7660757

