# Supplementary material for: Feasibility of Utilizing Telehealth in a Multidisciplinary Postpartum Hypertension Clinic
Source: Womens Health Rep (New Rochelle). 2022 Nov 1;3(1):877–86. doi: 10.1089/whr.2022.0066 (PMC9712043; doi:10.1089/whr.2022.0066)
Supplement: Supplemental data [file Suppl_Data.pdf]

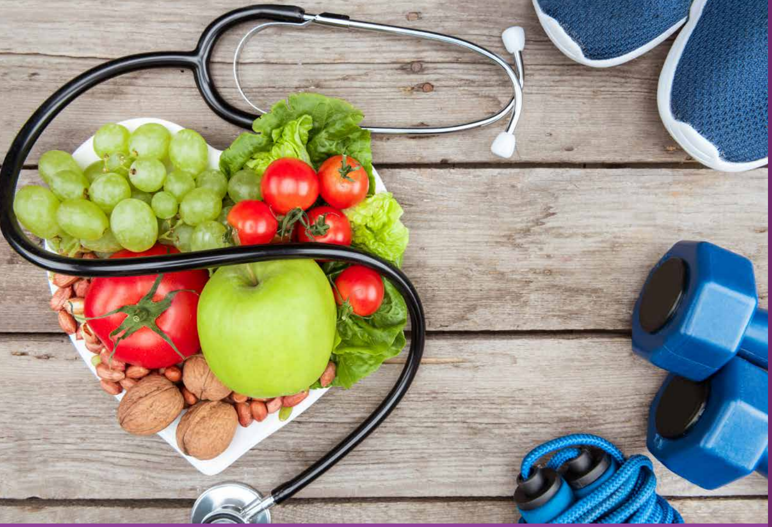

What you can do

Before and after delivering, here are two important ways you can control and manage your heart health:

Adopt a heart-healthy lifestyle: Eat a healthy diet, be more active, and try to maintain a healthy weight

Know your numbers: Get regular screenings for risk factors like high blood pressure, high cholesterol, and diabetes

UPMC
LIFE CHANGING MEDICINE

Postpartum Hypertension Program
UPMC Magee-Womens Hospital
Cardiology Department
300 Halket St., Suite 5102
Pittsburgh, PA 15213
Phone: 412-641-8870, option 0

UPMC policy prohibits discrimination or harassment on the basis of race, color, religion, ancestry, national origin, age, sex, genetics, sexual orientation, gender identity, marital status, familial status, disability, veteran status, or any other legally protected group status. Further, UPMC will continue to support and promote equal employment opportunity, human dignity, and racial, ethnic, and cultural diversity. This policy applies to admissions, employment, and access to and treatment in UPMC programs and activities. This commitment is made by UPMC in accordance with federal, state, and/or local laws and regulations.

MWH515042 JZ/MK 2/20

© 2020 UPMC

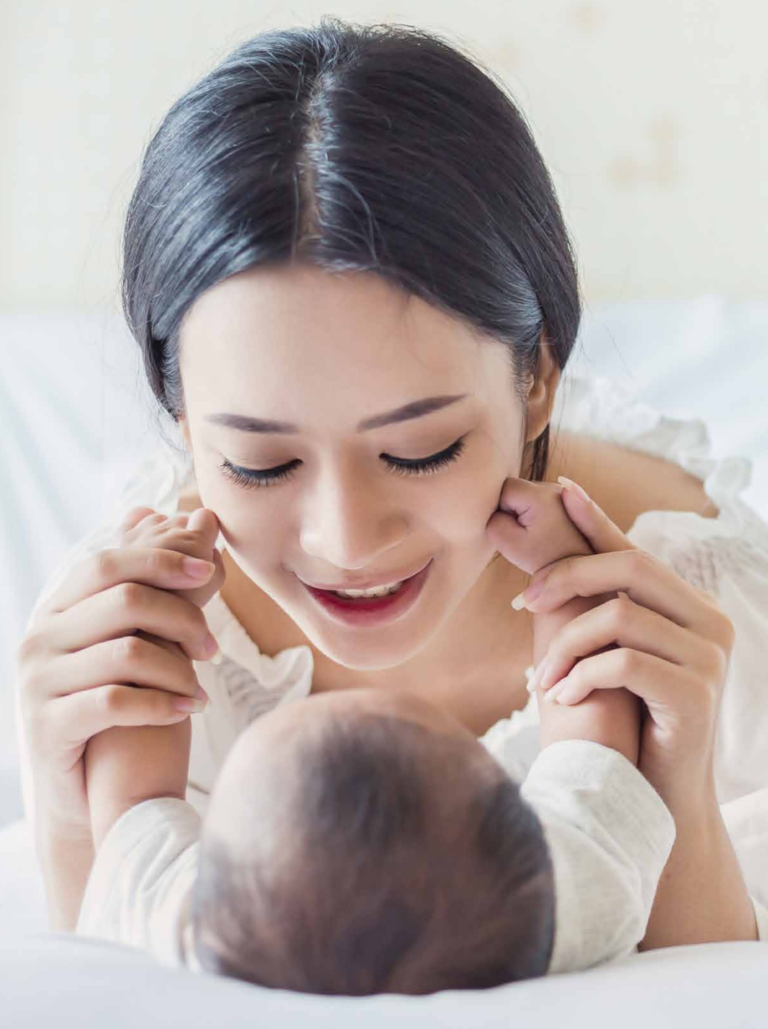

Postpartum Hypertension Program

Bringing together cardiology and maternal fetal medicine experts to help new moms stay heart healthy for life.

UPMC | MAGEE-WOMENS HOSPITAL

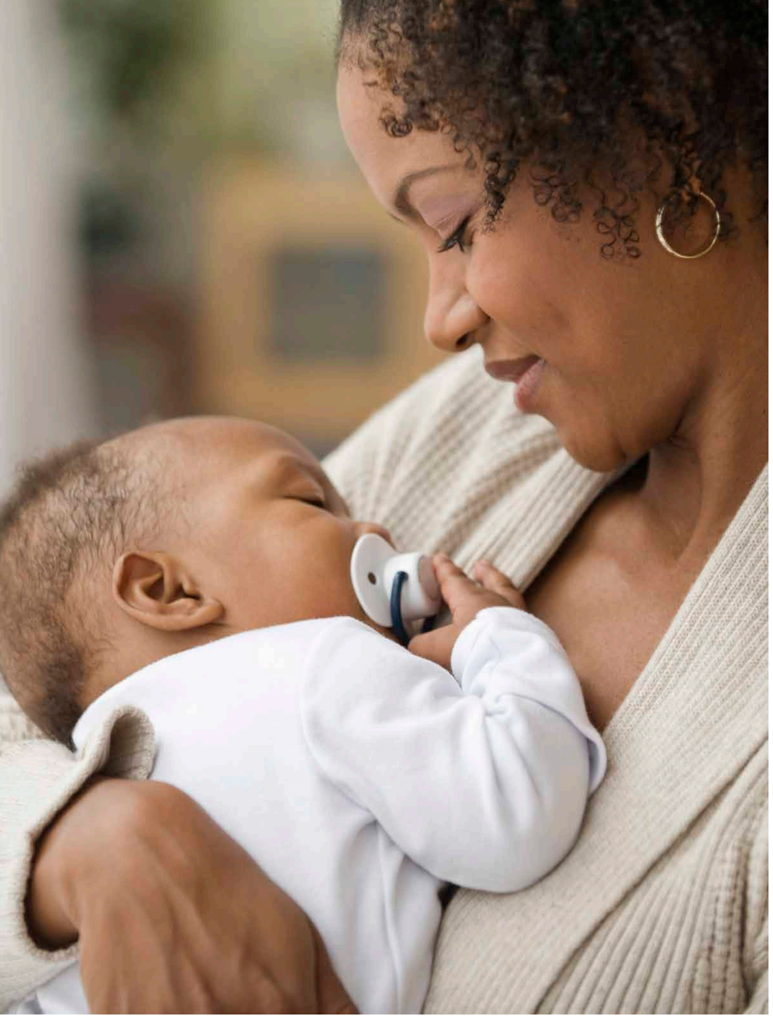

What is postpartum hypertension?

It's very common for new mothers to develop high blood pressure after delivery, especially if they experienced it when pregnant.

If you have high blood pressure (preeclampsia or gestational hypertension) during pregnancy, you are three to four times more likely to have it after delivery — as well as have a higher risk of future heart disease.

But with the right medical care, your high blood pressure can be treated. That's good news for you and your baby.

How we can help

A woman's heart goes through many changes during and after pregnancy. When you're pregnant, your heart has to work extra hard. You create and pump more blood for your baby. After delivery, your body has to adjust to many changes, including those affecting your heart.

Problems with high blood pressure can start during pregnancy or up to six months after having your baby. Without the right medical care, high blood pressure can lead to serious problems like heart disease and stroke. It can even be life threatening.

Located in the Cardiology Department at UPMC Magee-Womens Hospital, our Postpartum Hypertension Program includes experts in both women's heart health (cardiology) and high-risk pregnancies (maternal fetal medicine). These doctors have a special interest in helping women monitor and manage their high blood pressure after pregnancy.

Our services include:

- Screenings to determine your future heart risk
- Care to manage your high blood pressure
- Consultations for at-risk individuals who are considering becoming pregnant

Meet our experts

Cardiology

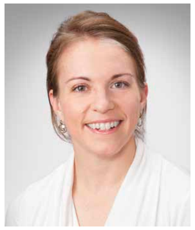

Kathryn Berlacher, MD

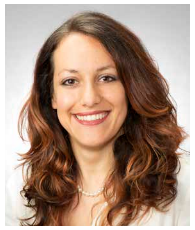

Malamo Countouris, MD

Maternal Fetal Medicine

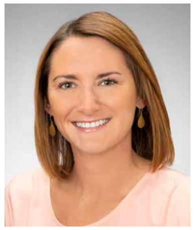

Alisse Hauspurg, MD

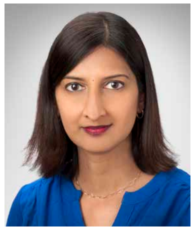

Arun Jeyabalan, MD

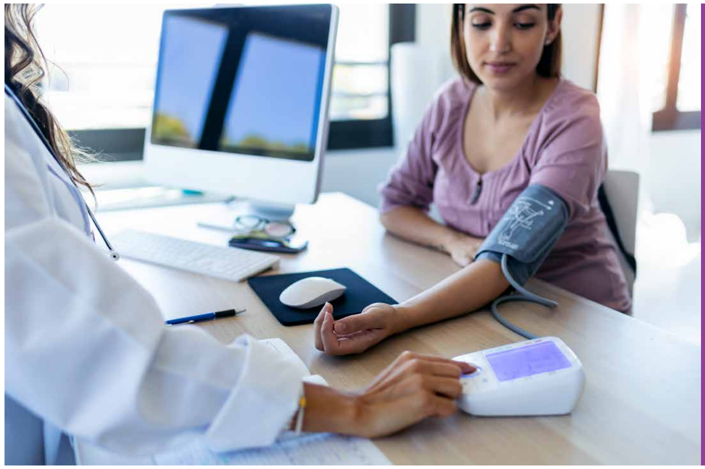

Talk to your doctors

Be sure to let all your doctors know about your pregnancy history.

Contact us

You or your doctor can request an appointment with our Postpartum Hypertension Program by calling 412-641-8870, option 0.
